# Small scale, elevation- and environmental-related differences in life history strategies in a temperate resident songbird

**DOI:** 10.1098/rsos.241777

**Published:** 2025-04-02

**Authors:** Benjamin R. Sonnenberg, Carrie L. Branch, Angela M. Pitera, Virginia K. Heinen, Lauren E. Whitenack, Joseph F. Welklin, Vladimir V. Pravosudov

**Affiliations:** ^1^ Department of Biology, University of Nevada Reno, Reno, NV, USA; ^2^ Department of Integrative Biology, Oregon State University, Corvallis, OR, USA; ^3^ University of Western Ontario, London, Ontario, Canada

**Keywords:** life history strategy, demographic tactics, reproductive variation, chickadees, elevation, environmental differences

## Abstract

Environmental drivers of within-population reproductive patterns are often hypothesized to lead to reproductive strategies tuned to local conditions. Organisms adjust energy allocation between survival and reproduction based on experience, age, lifespan and resource availability. Variation in these energetic investments can be described as different demographic tactics which are expected to optimize the fitness of local populations. These ideas are largely supported by both empirical and model-based studies but research identifying specific strategies and their corresponding environmental drivers within wild populations remains rare. Using 12 years of data, we investigated reproductive investment strategies in a relatively short-lived resident songbird, the mountain chickadee (*Poecile gambeli*), at two elevations that differ in environmental harshness in the North American Sierra Nevada mountains. Challenging winter environments at high elevations impose strong selection pressure on survival-related traits (e.g. specialized spatial cognition associated with food caching) and significantly shorten the length of the reproductive window. Here, we show that chickadees at a higher elevation lay smaller clutches (*ca* 0.41 fewer eggs) and produce fewer (*ca* 0.25 fewer nestlings) but larger offspring (*ca* 0.4 g heavier) compared to lower elevation residents. Due to the harsher and less predictable environmental conditions at higher elevations, this investment strategy in this resident species likely leads to the production of offspring with greater chances of survival. Overall, our results show that within-species differences in life history strategies may evolve over a small spatial scale along strong environmental gradients.

## 1. Introduction

Free-living animals fall along a wide spectrum of life history strategies, the outcomes of which are often shaped by local environments [1–5]. Such strategies are defined by differences in lifespan, survival rate, reproductive investment and the management of energetic costs to maintain homeostasis and cope with unpredictable events [1–4,6]. However, establishing the causes and consequences of variation both within and between populations of varying life history strategies remains challenging.

Classical theory on life history strategy explains large-scale patterns observed among species across time [3,4], but the drivers (e.g. environmental gradients associated with resource availability or climate) of within-population variation in life history strategies often remain context specific and elusive. One suite of potentially important drivers of within-species variation in life history strategies is the wide assortment of environmental conditions that wild populations experience across their ranges [2,7]. This includes both predictable and unpredictable climate patterns which may influence resource availability and even predation pressures [2,7,8]. All organisms must balance the costs of survival and individual maintenance with reproduction, and longer-lived organisms are expected to delay reproductive investment in challenging years, such as those with anomalous weather events, as these delays have little negative effect on their lifetime fitness [2–4,7]. Short-lived animals are expected to be disproportionally affected by similar challenges [3,4,7]. In fact, environmental perturbations may significantly drive the observed patterns of reproductive output in the wild populations of short-lived organisms across and within years [3,4,7].

Avian species are ideal models for testing how environmental variation can influence differences in life history strategies as birds exhibit a wide range of tactics and their reproductive output can be readily measured. For example, clutch size (i.e. total number of eggs an individual lays per reproductive bout) and brood size (total number of offspring that successfully result from a clutch) have been measured in hundreds of species and have been of interest for decades [9–12]. Egg production is energetically costly and clutch sizes have been shown to vary with latitude, elevation, nest type, avian body size, seasonal timing, parent age and developmental mode of the offspring [12–16]. For example, short-lived and smaller-bodied species consistently reach sexual maturity faster and produce more offspring per attempt throughout their life, compared to longer-lived and larger-bodied species [3,11,17].

Limitations on clutch size have long been hypothesized to be related to local conditions and the physiological condition of individual females [9]. This hypothesis has mixed support; while clutch size shows a clear relationship with environment (e.g. resource availability, latitude or elevation) in some species [18–23], other examples suggest that variation in clutch size is heritable, as it shows within-individual repeatability within populations across diverse resource availability [11,24]. Clutch size and brood size are often highly correlated as the number of offspring that parents care for is highly tied to egg number, local environmental factors and yearly variation (e.g. food abundance and weather events) [2,11]. Thus, individuals must bear costs across the entire reproductive period to maximize lifetime fitness. Such costs likely generate tradeoffs between producing the maximum number of eggs, the subsequent care of the resulting offspring and quality of these offspring in some species [11,25]. These reproductive tradeoffs are not well understood in environments showing wide ranges of climatic severity, including high montane elevations that experience large differences in cold temperature and high snow levels [15,25]. Elevation gradients provide the opportunity for natural environmental comparisons, as high elevations are associated with colder temperatures, shorter reproductive periods, decreased partial pressure of atmospheric gases (i.e. influencing oxygen availability to eggs), and less reliable food availability compared to lower elevations [12,26]. High elevation environments in temperate montane forests show higher numbers of unpredictable weather events which could limit access to resources and induce costly oxidative stress responses on both parents and offspring [22,26,27]. Taken together, this evidence suggests that higher elevations exhibit harsher ecological conditions.

The reproductive reduction hypothesis predicts that individuals who reproduce at higher elevations will produce fewer offspring but invest more into those offspring in response to challenging harsh environments [12,15,28]. There is mixed evidence for elevation-related patterns of reproductive output, but the majority of avian species in temperate and tropical climates show smaller clutches at higher elevations [12,18,20,21,28,29]. In addition to smaller clutch sizes, most of these species exhibit increased parental care (i.e. provisioning visits), supporting the reduction hypothesis, even though these same studies do not show that this increased care translated into higher quality offspring [12,15,18]. These previous studies mainly considered migratory species that are required to assess and adjust to local conditions after long-distance movement immediately prior to reproduction [11,15,28,29]. Furthermore, most studies compared closely related taxa (i.e. across species comparisons) across elevation gradients and not within-population variation of the same species inhabiting heterogenous gradients [11,18,30]. Thus, it is less clear whether the variation found is associated with within-species life history strategies driven by environmental conditions or species differences.

We studied reproductive patterns in a resident population of food-caching mountain chickadees (*Poecile gambeli*) in the Sierra Nevada mountains, USA, across 12 years and two elevation sites. These sites are separated by a snow line [31–33], where higher elevations above this relative altitude have a larger and longer lasting snowpack, that is accompanied with cooler temperatures compared to the lower elevations [22,26,34]. Moreover, environmental conditions at this location do not change gradually with elevation but change abruptly near the snow line [22,26,33,34]. This allows for comparisons between elevation-related sites that are associated with large environmental, climate-related differences [22,26,33,34]. This system has served as a model to study adaptations to harsh environmental conditions for more than a decade [35,36]. Mountain chickadees are short-lived passerine songbirds (average lifespan of a bird that survives until its first autumn is ~2 years; more information provided in Methods [37]) and reside year-round in coniferous montane environments in western North America [38]. Residing in such environments year-round makes this species particularly susceptible to adverse local climatic conditions. Higher elevations are associated with large swings in yearly snow depth which often persist into the summer months. In years with deep, continuing snow, chickadees have limited access to nest sites and have a shorter reproductive window [22,26,34]. Regardless of yearly variation in snow conditions, the higher elevation site consistently has more snow and cooler temperatures compared to lower elevations [26,34]. Chickadees in this system are highly resident and show no elevational movement after a short-distance postnatal dispersal [38,39] (personal observations by the authors), but there is no clear population structure which suggests gene flow between elevations [40]. This gene flow may be limited via behavioural mechanisms as individuals sing different songs between elevations and higher elevation dwelling females prefer males from their same elevation in pair-wise captive tests [41,42]. Chickadees exhibit scatter food-hoarding behaviour; individuals store tens of thousands of caches and rely on these caches to survive winter [36,38,43]. Harsh winter conditions are associated with stronger selection on survival-related traits [44,45], and previous work has shown that chickadees inhabiting higher elevations in this system store more food [35] and have better spatial cognitive abilities [35,46,47] associated with larger hippocampi (i.e. area of the brain associated with spatial memory), including more and larger neurons [35,48]. The variation in these morphological and cognitive differences is associated with large genetic differences and is highly heritable [49,50]. In addition, higher elevation birds with better spatial learning and memory abilities are more likely to survive their first winter compared to other members of their cohort with worse performance and show higher longevity overall [37,45]. This previous body of work shows that differences in environmental conditions associated with winter climate severity can result in local adaptation of complex traits (i.e. related to food caching and spatial memory) in the face of gene flow within a single population [36,40,45,49]. Taking these results into account, we suggest that producing fewer, higher quality offspring that are more likely to survive the first winter selection event at higher elevations may be a favourable strategy within this population.

Here, we tested whether environmental differences between high and low elevations were associated with different life history strategies, focusing on reproductive output (i.e. clutch size, brood size, mean nestling mass and brood reduction). Considering that birds at high elevation encounter harsher environments [22,26], show higher adult survival rates [51], and lower juvenile recruitment compared to birds at low elevation [52], we expected that chickadees at the two elevations engage in different reproductive strategies. Additionally, chickadees must invest in energetically expensive neural tissues (i.e. the hippocampus) that are required for memory storage for successful food-cache recovery and overwinter survival [36,53]. We predicted that chickadees at high elevation lay smaller clutches and consequently produce smaller broods. At the same time, we predicted that high elevation chickadees invest more in parental care and fledge higher quality offspring relative to brood size, compared to low elevation birds. Producing fewer but higher quality offspring may increase the likelihood of successful recruitment in harsher environmental conditions [28]. Alternatively, it may be that these animals are not exhibiting separate life history strategies but are plastically responding to conditions year to year. If they adjust reproduction based on conditions within a given year, we would expect similar reductions in clutch sizes and brood sizes in suboptimal conditions regardless of elevation. If this were the case, high elevation chickadees would produce fewer young of similar or worse quality than those at lower elevations. If chickadees are indeed exhibiting different life history strategies, we would see a tradeoff: either producing more offspring of lower quality or fewer of higher quality. Thus, fledgling conditions provide a means to discriminate between these two alternatives. We used fledgling body mass as an index of offspring quality because it is well known to be positively associated with survival and recruitment [28,54] and we have shown this directly in this system [39]. Overall, we predicted that high elevation birds invest less in the production of offspring (i.e. smaller clutch size) but more in subsequent offspring care (i.e. incubation and provisioning) resulting in higher quality offspring compared to low elevation birds. This would provide novel evidence of within-population elevation-related reproductive strategy differences across a small spatial scale. In this study, we test this idea by investigating 12 years of clutch size, brood size, mean nestling mass and brood reduction data from a long-term mountain chickadee population monitored in the Sierra Nevada mountains.

## 2. Methods

### 2.1. Subjects and study site

**Figure 1.**
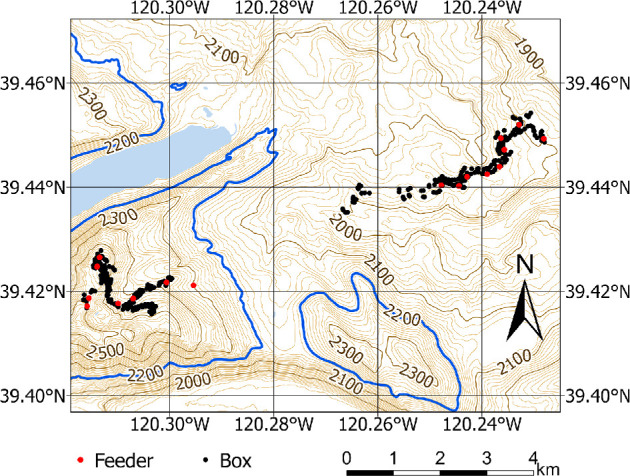
Map of the entire field site including locations of all nest boxes and feeders where trapping and banding occurred during the study.

All data for this study were collected between April 2013 and August 2024 (representing 12 chickadee breeding seasons) at Sagehen Experimental Forest (Sagehen Creek Field Station, Tahoe National Forest, University of California, Berkeley, CA, USA) (figure 1). This population was monitored at two elevations separated by approximately 3.49 km, ‘high’ (range: 2380– 2590 m; coordinates: 39.42402, −120.315015) and ‘low’ (range: 1965–2070 m; coordinates: 39.443500, −120.243248) where we maintained *ca* 18 bird feeders during autumn and winter and *ca* 350 nest boxes during the spring and summer [22,26,55]. Low elevation boxes and feeders stretch along ~6.5 km of United States Forest Service roads and high elevation along ~3.6 km. There is not less box occupancy at the high elevation site despite differences in sample size. Sample differences are due to fewer boxes at high elevation as the site sits across a saddle of a ridgeline which provides less physical area for box placement. The entire forest (i.e. both elevation sites) are characterized by highly homogeneous coniferous forest composed of a variety of pine species (e.g. Jeffrey pine (*Pinus jeffreyi*), ponderosa pine (*Pinus ponderosa*), lodgepole pine (*Pinus contorta*), sugar pine (*Pinus lambertiana*), white pine (*Pinus monticola*), mountain hemlock (*Tsuga mertensiana*), and red (*Abies magnifica*) and white (*Abies concolor*) firs) and similar chickadee density.

Chickadees were systemically trapped across years in the autumn and winter months (August–March) using mist nets at established feeders and during the spring and summer months (May–July) by hand in nest boxes during reproductive monitoring. Birds were individually marked with unique combinations of coloured plastic leg bands, individually numbered aluminium bands issued by the United States Geological Survey (USGS), and coloured leg bands with imbedded passive integrated transponders (PIT tags). This marking methodology allowed for identification of unique individuals by sight, in the hand when recaptured, or when birds visited nest boxes or feeders equipped with radio-frequency identification technology (RFID) throughout the year [14,56,57]. Upon initial capture, all birds were aged and sexed when possible, using a combination of physiological (sex: brood patch, cloacal protuberance) and morphological (age: feather structure and molt pattern) indicators [58]. This intensive monitoring allowed for calculations of the average longevity of adults (birds that survive until their first autumn) ~2 years (*n* = 227 birds; mean lifespan = 2.1 years; median lifespan = 2 years; max. lifespan = 8 years; min. = 0 years; s.d. = 1.8 years) [37]. This means that the average bird in this population only reproduces twice during its lifetime and so the difference between producing one or two more eggs per season has substantial consequences for lifetime reproductive success. This analysis does not consider animals tracked from the egg or nestling phase and, if included, the average lifespan estimate would likely drop below 1 year of life. Either estimate suggests that chickadees are a short-lived species that must balance the costs of individual maintenance and reproduction.

### 2.2. Reproductive monitoring

Nest boxes were monitored for reproductive activity starting in early April of each calendar year. Monitoring consisted of weekly visits to each box to detect nest building and egg laying [22,26,34,59]. Once an egg was detected, visits increased in frequency (approx. every other day) to accurately detect the date of incubation initiation. Clutch size (total number of eggs laid per nest) was determined once incubation started. Chickadees incubate for approximately 13 days [38] (our personal unpublished data) which allowed for precise determination and detection of hatch date (nests were checked for hatching every other day after 12 days of incubation). All nestlings were processed at 16 days post-hatch for accurate across- and within-year comparisons. Hatch is recorded as day 1 in this system as opposed to day 0. During nestling processing, all nestlings were removed from a nest box and counted to determine brood size (total number of nestlings alive at day 16 per nest) as the vast majority of nestlings present in the nest at this time successfully fledge (personal observations of the authors). Individuals were then marked with a single USGS aluminium band, weighed (to the nearest 0.01 g), and a blood sample (~100 µl) was taken from each nestling for genetic analyses. Brood reduction was determined by subtracting the brood size at day 16 from the clutch size. This metric is often related to local environmental factors such as resource availability, weather events that lead to increased thermoregulatory costs for the offspring, poor parental care effort or even siblicide [14,56,57]. In this system, it is likely limited to environmental challenges such as resource availability as no nest failure in this system has been linked to a cold snap or rainstorm event (personal observations of the authors). Only the first breeding attempt of each pair per year was used in all the following analysis. This banded population and close monitoring scheme allows birds attempting to renest after a depredation event to be easily detected. If unbanded birds were observed at a failed box, the nest data of unbanded birds attempting a new nest near this previous failure site were conservatively removed. Second clutches are rare in this system with approximately 10 being recorded across the entire 12 years of study and only at the low elevation site. These were also removed from the following analyses. Finally, anti-nest-predator defences (aluminium sheets surrounding tree trunks and placed under nest boxes) were deployed in this system. These preventative methods against nest predators, mainly mammalian rodents (e.g. chipmunks), were not fully effective and limit our ability to evaluate natural rates of predation in this system. We do not see this as a constraint as our main goal for this study was to investigate parental investment by using complete clutch, brood and nestling condition measurements. Overall, 1135 nests were used in analyses, 669 from low elevation and 466 from high elevation. Nests with known parentage totalled 871, 500 from low elevation and 371 from high elevation.

### 2.3. Statistical methods

We tested elevation-related differences in within-population patterns of reproductive output using clutch size, brood size, mean nestling mass and brood reduction as response variables and elevation as a fixed categorical predictor variable. For all analyses, we only included nests from the first breeding attempt of a given pair within a season. Mountain chickadees at this site rarely produce a second brood per year (and never at high elevation) or renest (if depredated late in the season [26]); therefore, these attempts were excluded from this study. As we were specifically testing for patterns of variation between elevations and not yearly variation, year was included as a random effect in all models (11 levels: 2013–2024). We ran models for each reproductive parameter twice, first including all samples within the dataset and second including male and female identity as random effects. This second iteration was performed to control for the effects of parental experiences as experienced pairs produce more and larger young in this population [60]. The second method utilized a smaller sample size, due in part to individual identification of breeding birds not being recorded in the first few years of the study.

All models and associated graphics were generated using R v. 4.3.1 [61,62]. We used linear mixed models to model the effect of elevation on measures of reproductive output (clutch size, brood size, mean nestling mass, and brood reduction). To model both clutch and brood size count data, we fitted models using the R package glmmTMB using truncated generalized Poisson or Conway–Maxwell distributions as these data lack zeros [63,64]. Mean nestling mass was modelled with a Gaussian distribution and brood reduction with a negative binomial distribution. As nestlings of smaller broods can be expected to be heavier than larger broods at the same level of parental investment, we investigated fledgling mass relative to brood size by using brood size as a covariate. We report models both with and without this covariate. *Post hoc* analyses used to calculate marginal means were conducted with the emmeans package [65]. Model fits were confirmed using the DHARMa package [66].

## 3. Results

### 3.1. Clutch size

**Figure 2.**
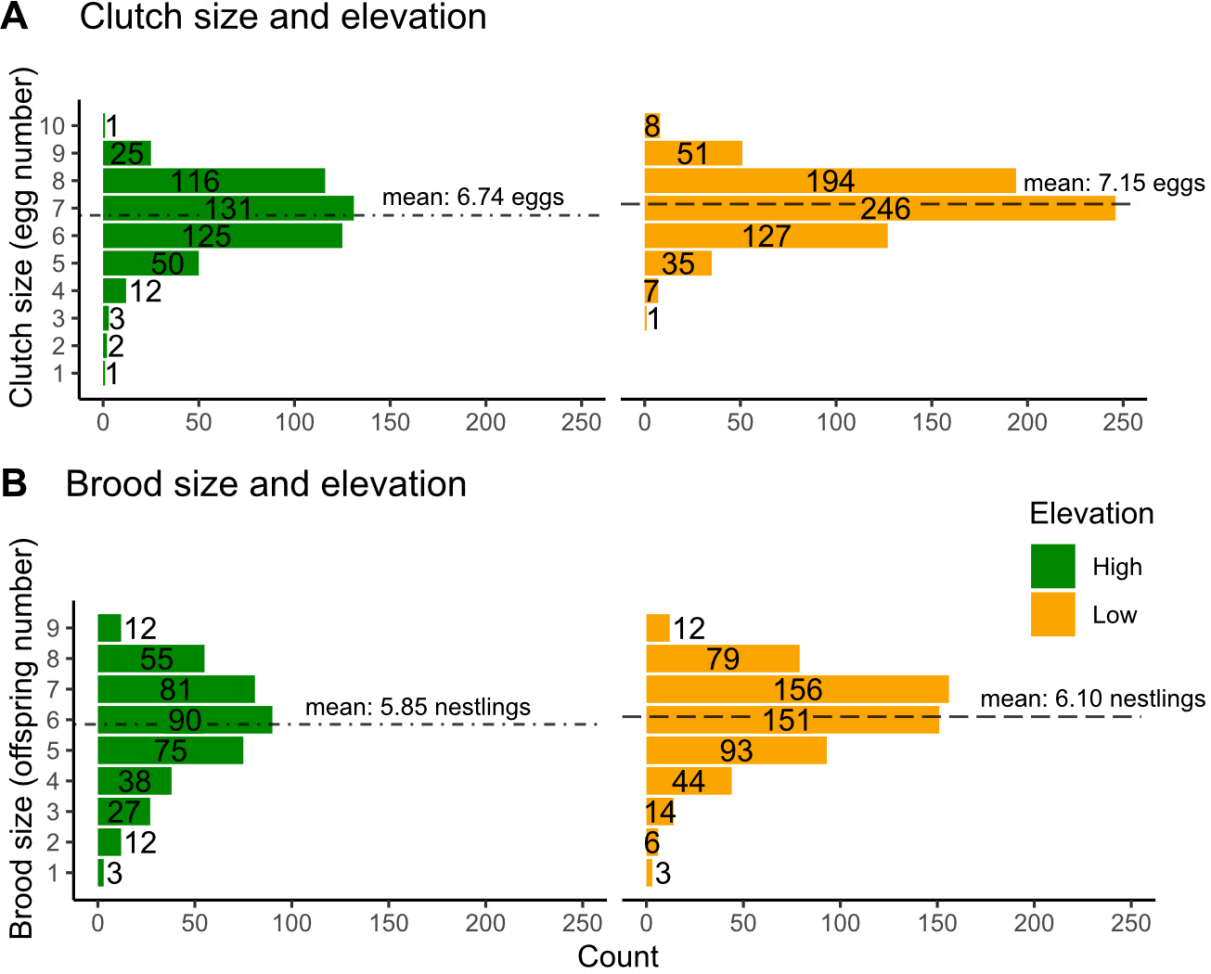
Clutch (A) and brood (B) sizes across elevations (high elevation marked in green and low elevation marked in orange) including frequency plots of each metric with dashed lines representing marginal means.

Chickadees at low elevation laid larger clutches compared to birds at high elevation (table 1 and figure 2). A *post hoc* analysis conducted using the less conservative dataset (the entire dataset, including parental birds with unknown identification) showed that on average chickadees at low elevation laid 0.41 more eggs (table 1; model 1: marginal means: low: 7.15 ± 0.1 eggs, *n* = 669; high: 6.74 ± 0.1 eggs, *n* = 466; *post hoc* model: ratio = 0.94 ± 0.01, *z* = −6.01, *p* < 0.001). This result was qualitatively the same for the more conservative analysis that contained only known pairs (model 2; table 1), but there was an even more striking difference in clutch size between elevations using the more conservative model (marginal means: low: 7.23 ± 0.1 eggs, *n* = 500; high: 6.71 ± 0.1 eggs, *n* = 371; *post hoc* model: ratio = 0.93 ± 0.01, *z* = −5.87, *p* < 0.001).

**Table 1.** Linear models testing for differences in clutch size between elevation sites; model 1 contains the entire dataset and model 2 controls for effects of known individuals.

fixed effect	*N*	parameter estimate	s.e.	*z*-value	*p*-value
model 1: clutch size differences between elevations using full dataset					
intercept	1135	1.91	0.02	100.76	*p* < 0.001***
elevation		0.06	0.01	6.01	*p* < 0.001***
mixed effect variances: year: 0.003 ± 0.06 AIC: 3525.2					

model 2: clutch size differences between elevations using nests of known parents					
intercept	871	1.90	0.02	91.96	*p* < 0.001***
elevation		0.07	0.01	5.87	*p* < 0.001***
mixed effect variances: year: 0.003 ± 0.06 female identification: 4.2 × 10^−10^ ± 2.1 × 10^−5^ male identification: 7.1 × 10^−3^ ± 8.4 × 10^−2^ AIC: 2578.6					

### 3.2. Brood size

Chickadees at low elevation had larger brood sizes compared to birds at high elevation (table 2 and figure 2). The less conservative model (marginal means: low elevation: 6.1 ± 0.1 nestlings, *n* = 558; high elevation: 5.85 ± 0.1 nestlings, *n* = 393; *post hoc* model: ratio = 0.96 ± 0.01, *z* = −2.64, *p* < 0.01; table 2: model 1) and the more conservative model including only known individuals (marginal means: low elevation: 6.22 ± 0.1 nestlings, *n* = 464; high elevation: 5.89 ± 0.1 nestlings, *n* = 336; *post hoc* model: ratio = 0.95 ± 0.02, *z* = −3.03, *p* < 0.01) showed similar results. Brood size at low elevation was larger by 0.25–0.33 offspring in both models.

**Table 2.** Linear models testing for differences in brood size between elevation sites; model 1 contains the entire dataset and model 2 controls for effects of known individuals.

fixed effect	*N*	parameter estimate	s.e.	*z*-value	*p*-value
model 1: brood size differences between elevations using full dataset					
intercept	951	1.77	0.02	82.10	*p* < 0.001***
elevation		0.04	0.01	2.65	*p* < 0.01**
mixed effect effects variance: year: 0.004 ± 0.06 AIC: 3486.4					

model 2: brood size differences between elevations using nests of known parents					
intercept	800	1.77	0.02	73.83	*p* < 0.001***
elevation		0.05	0.02	3.03	*p* < 0.01**
mixed effect variances: year: 0.004 ± 0.06 female identification: 0.002 ± 0.05 male identification: 0.004 ± 0.06 AIC: 2879.2					

### 3.3. Mean nestling mass and brood reduction

**Figure 3.**
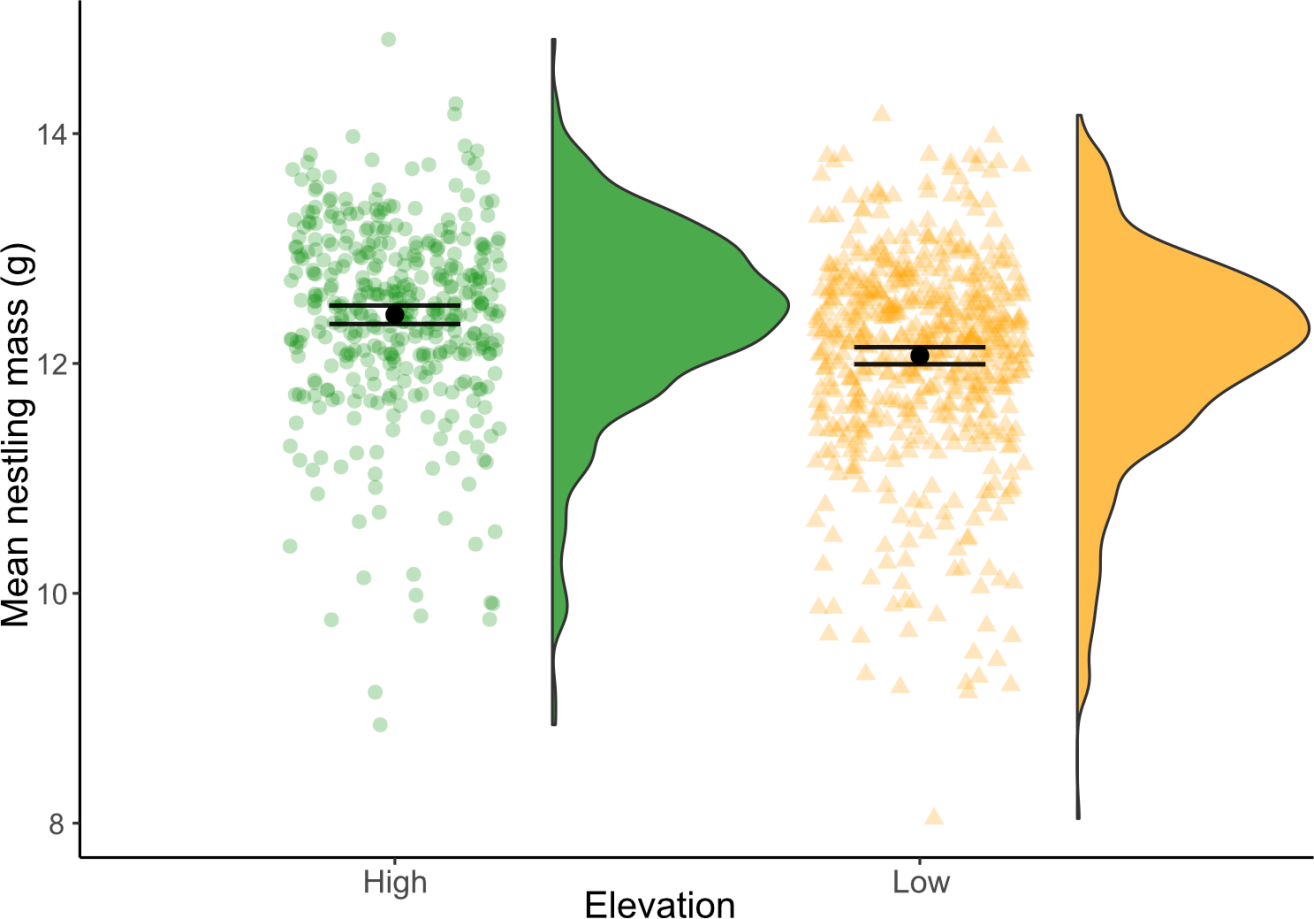
Mean nestling mass with 95% confidence intervals by elevation.

Chickadees at high elevation fledged young with higher body mass compared to birds at low elevations (table 3 and figure 3). A *post hoc* analysis from the less conservative model showed an estimated difference of 0.4 g between high and low elevations (marginal means: low elevation: 12.1 ± 0.1 g, *n* = 555; high elevation: 12.5 ± 0.1 g, *n* = 391; *post hoc* model: ratio = 0.36 ± 0.05, *t* = 6.70, *p* < 0.001). The more conservative model including only known individuals produced the same result (marginal means: low elevation: 12.0 ± 0.1 g, *n* = 459; high elevation: 12.3 ± 0.1 g, *n* = 334; *post hoc* model: ratio = 0.34 ± 0.06, *t* = 5.36, *p* < 0.001). These results were nearly identical to models excluding brood size as a covariate (table 3). If anything, models including brood were a slightly better model fit according to AIC values (table 3). There were no differences in brood reduction between the elevations (table 4). This was the case in both sets of models.

**Table 3.** Linear models testing for differences in nestling mass between elevation sites; models 1 and 3 contain the entire dataset and models 2 and 4 control for effects of known individuals. Models 1 and 2 contain brood size as a covariate while models 3 and 4 do not.

fixed effect	*N*	parameter estimate	s.e.	*z*-value	*p*-value
model 1: nestling mass differences between elevations using full dataset					
intercept	946	12.56	0.15	85.27	*p* < 0.001***
elevation		−0.35	0.05	−6.70	*p* < 0.001**
brood size		−0.02	0.02	−1.01	*p* = 0.31
mixed effect variance: year: 0.12 ± 0.35 AIC: 2265.9					

model 2: nestling mass differences between elevations using nests of known parents					
intercept	793	12.48	0.16	78.09	*p* < 0.001***
elevation		−0.34	0.06	−5.36	*p* < 0.001**
brood size		−0.02	0.02	−1.18	*p* = 0.24
mixed effect variances: year: 0.11 ± 0.33 female identification: 0.06 ± 0.24 male identification: 0.02 ± 0.15 AIC: 1873.9					

model 3: nestling mass differences between elevations without brood size using full dataset				
intercept	946	12.45	0.11	*p* < 0.001***
elevation		−0.36	0.05	*p* < 0.001***
mixed effect variances: year: 0.12 ± 0.35 AIC: 2271.1				

model 4: nestling mass differences between elevations without brood size using known parents				
intercept	793	12.35	0.11	*p* < 0.001***
elevation		−0.35	0.06	*p* < 0.001***
mixed effect variances: year: 0.10 ± 0.32 female identification: 0.06 ± 0.24 male identification: 0.02 ± 0.14 AIC: 1874.8				

**Table 4.** Linear models testing for differences in brood reduction between elevation sites; model 1 contains the entire dataset and model 2 controls for effects of known individuals.

fixed effect	*N*	parameter estimate	s.e.	*z*-value	*p*-value
model 1: brood reduction differences between elevations using full dataset					
intercept	951	−0.008	0.07	−0.11	*p* = 0.91
elevation		0.06	0.07	0.82	*p* = 0.41
mixed effect variance: year: 0.02 ± 0.13 AIC: 2634.6					

model 2: brood reduction differences between elevations using nests of known parents					
intercept	800	−0.11	0.09	−1.30	*p* = 0.19
elevation		0.09	0.09	0.98	*p* = 0.33
mixed effect variance: year: 0.016 ± 0.1 female: 0.03 ± 0.18 male: 0.05 ± 0.21 AIC: 2170.1					

## 4. Discussion

By comparing several metrics of reproductive output in chickadees inhabiting high versus low elevations, we found that chickadees at low elevation produced larger clutch and brood sizes but chickadees at higher elevations produced fewer but higher quality (as indicated by larger body mass) offspring. These results demonstrate that highly resident chickadees living at higher elevations with harsher environmental conditions exhibit a different reproductive strategy from birds at lower elevations over a small spatial scale (<8 km). While it could be expected that smaller broods in this context would always produce young of higher quality, higher elevation nests consistently produced heavier nestlings independent of brood size. These results show a classic tradeoff in reproductive investment between number of offspring and offspring quality, indicating support for two separate elevation-based life history strategies within a resident temperate species. However, this study was only conducted within a single population and at two sampling locations that differed in elevation, which severely limits the broad applicability of these results to other montane resident species across comparable elevation differences.

These reproductive differences suggest the presence of two strategies that are distinct from a plastic response to variation in local environmental conditions [9]. If we had not found evidence for our initial predictions, we would have concluded that the observed variation in reproductive output is due to a plastic local response to yearly environmental variation. If chickadees responded directly to local environments associated with each elevation, we would expect birds in poor environments (i.e. less resources available for both parents and offspring) to lay smaller clutches because of direct environmental limitations [28]. If that were the case, we would not expect that birds with smaller clutches would rear higher quality young. Instead, we would predict that nests with varying clutch sizes would produce young of similar quality and individual parents (i.e. parental quality) would be the best predictor of clutch size and subsequent offspring quality. Despite this prediction, we found that high elevation nests had smaller clutch and brood sizes but larger offspring. In addition, past results from this system show that supplemental food during winter, predicted to enhance the physical condition of parents (i.e. parental quality) entering the nesting season, had no detectable effect on timing of breeding, clutch or brood size or nestling mass [59]. This suggests that despite consistent exposure to supplemental resources prior to reproduction, birds still showed different but consistent investment patterns between elevations (i.e. larger clutches and smaller offspring at low elevation, smaller clutches and larger offspring at high elevation).

We observed no differences in brood reduction between elevations. The raw averages for each elevation site were quite low (high = 1.01 and low = 1.04). This was opposite to our prediction but still supports the presence of separate reproductive strategies. If the differences in brood size were driven by brood reduction (due to nestling mortality or unhatched eggs), this would suggest that higher elevation birds were laying more eggs than they could successfully fledge or laying more inviable eggs (i.e. suggesting mate asynchrony or impacts to parental incubation effort or local weather such as rainstorms impacting egg development) [56,57], which appears not to be the case for this population. The fact that this population shows no difference in brood reduction suggests that there may be similar resource availability at each elevation site, and that even in poor resource years the pattern of high elevation parents investing more in their young post hatch remains true. Additionally, this consistent low brood reduction does not add support to reproductive investment being highly plastic in this species. These consistent results support the idea that these patterns may have evolved via different selection pressures due to large environmental differences between elevations at this site.

At high elevations, producing fewer but heavier (i.e. higher quality) young has been predicted to lead to higher survival rates for offspring in these harsh conditions for avian species [12,15,18,28]. Past work in our system showed higher annual survival of adults at higher elevations compared to adults at lower elevations [51,52]. At lower elevations with milder climates associated with relaxed selection pressure, producing more, lower quality young likely results in higher overall fitness, while larger investment in a small number of young at higher elevation may optimize fitness when environmental conditions are harsh especially during the first winter as this is the major selection event for individuals recruiting into the population [45]. As the average lifespan of a recruited adult in this population is only 2 years, a difference of half an egg has significant implications for lifetime reproductive success for individuals. Our evidence suggests that individuals coping with harsher conditions (i.e. high elevation birds) are making this investment in offspring care over producing more eggs in these conditions.

Chickadees are resident species in western North America and primarily reside in montane regions after a short-distance postnatal dispersal [38] (and personal observations from this system). They are scatter-hoarders that cache tens of thousands of food items every autumn and early winter and rely on specialized spatial cognitive abilities (i.e. spatial learning and memory ability) to recover these caches to survive not only their first winter but throughout their lives [36,37,45]. These traits are fundamental to the life history of mountain chickadees and have been shown to have a strong genetic basis, to be under directional natural selection and to predict longevity, particularly at high elevations [37,45,49,50]. However, cognitive traits are energetically expensive and higher parental investment during development may be critical for chickadee offspring [36,53]. These adaptive cognitive traits may drive differential investment in high elevation birds as producing larger offspring could be aiding their cognitive development, as well as improving their overall condition before their first winter, and increasing their odds of survival. While there is gene flow between elevations [40], it is known that strong selection can produce evolutionary changes in the presence of gene flow, resulting in the evolution of local adaptations [67,68].

The prediction that producing larger, higher quality nestlings at high elevation is related to future food-caching behaviours and their underlying cognitive abilities are supported by the foundational work in this system [22,35–37,44–46,48,49,69]. However, there may be alternative explanations for these patterns. Predation, especially during the immediate post-fledging state, could be another potential evolutionary driver for this pattern. Nestlings that leave the nest at higher mass may have a better immediate chance of avoiding predators. Although this would likely only be the case if there were differences in predator densities between elevations at the focal area of study. Unfortunately, we have no direct evidence for this as the main predators of fledgling chickadees (e.g. sharp-shinned hawks (*Accipter striatus*) and northern pygmy-owls (*Glaucidium gnoma*)) population densities have never been measured as part of the current long-term study. Past survey evidence from before the long-term study suggests that both species occur at very low densities in the Sierra Nevada which does corroborate personal observations of all the authors involved in this study [70–72]. As such we have no evidence that the density of predators can differ between elevations. Additionally, there are very few of these predators detected during nest surveys and there have never been nearby raptor nests discovered close to the box transect lines at either elevation site during the time of the study (personal observations of the authors). This is quite different from European tit species which seem to inhabit forests with higher aerial predator densities, but this shows the importance of studying multiple species across diverse habitats [73–77]. The main predation pressure during the nesting period for chickadees comes directly from local rodent species and depredation events occur before fledgling in these cases (personal observations of the authors) [38]. Thus, it is unlikely that predation pressures during or after the nestling periods are the main evolutionary drivers between the observed differences in reproductive investment between elevations. Nevertheless, it cannot be completely ruled out without further study.

Understanding the drivers of variation in avian reproductive output across broad spatial scales, both within and between species, has been of interest for nearly 100 years [11]. Much of the variation between species can be attributed to classical life history strategies such as K versus r selection [4,11]. However, differences within populations are often thought to be driven by parental experience or local resource availability rather than separate strategies [9]. This would suggest that these traits may be plastic within a species given their environment or the reproducing pair’s response to their local environment. Parental experience does affect chickadee populations, as we have shown that more experienced pairs produce more, and larger offspring compared to first-time breeders [60]. Our approach in the current study was to examine reproductive performance of the population subsets of each elevation, controlling for the effects of parental identity, and we still found a strong elevation-related difference in reproductive output. Additionally, extra pair paternity does occur in this system (unpublished data) but chickadee males do not exhibit parental care to offspring outside of their social partners nest (known from nest monitoring data from this system). Thus, promiscuity likely does not play a role in the observed differences in clutch size, brood size or mass in chickadees. Overall, this study shows that differences in reproductive strategies may arise over small spatial scales in the presence of strong environmental differences.

There has been controversy regarding differences in clutch size across elevations, with multiple compelling and competing hypotheses [10,28]. Higher elevations experience more unpredictable weather which could limit females’ access to resources during egg production, incubation or offspring provisioning. Observations in European populations where high elevation dwellers show smaller clutch sizes, support this hypothesis [18]. An alternative hypothesis suggests that larger clutch sizes could aid in nestlings gaining thermoregulatory independence from their parents earlier in development fuelled by more offspring in the nest, which suggests larger clutches would be adaptive at colder, higher elevations [10]. While a pattern of larger clutch sizes at high elevations has been observed in a few North American avian species [20], many of these studies simply compared closely related taxa that reside at different elevations during reproduction, none of which reside across elevation gradients. In our study, we found smaller clutch sizes at high elevations but also showed that these high elevation offspring were of significantly higher mass. This study, although containing 12 years of data, was only completed at two elevations in a single location. This is a typical tradeoff between collecting fewer comprehensive data at multiple broad locations versus more detailed data at a single location. Thus, the patterns seen in this population may not have broad applicability to other resident montane species in North America or elsewhere. However, we do not think that this pattern is a response to elevation *per se* but to a strong environmental gradient. Our previous work shows that the main environmental feature affecting selection in these resident food-caching birds is winter climate harshness [36]. Differences in cognition and food caching are highly consistent across both elevation and continental-level scales in two different chickadee species [36], suggesting that these are important gradients to consider and may also be impacting complex reproductive patterns and behaviours on these same scales. Despite reporting differences in reproductive strategies just at this single site, we think that this is important work that will fuel questions concerning how populations may respond to future changes in local conditions especially when climate related.

**Figure 4.**
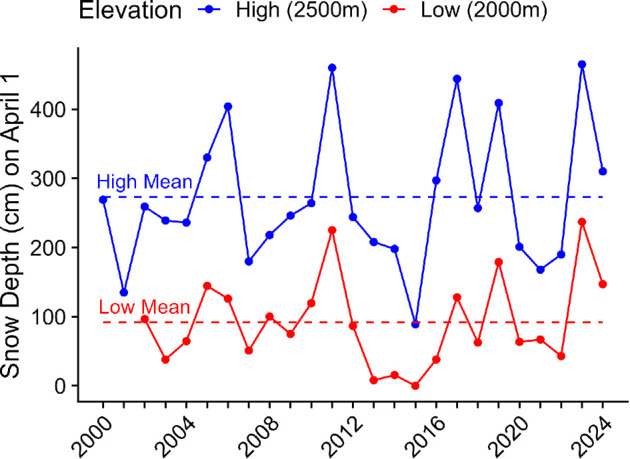
Mean differences in snow depth between elevations and across years of the study (high elevation marked in blue; low elevation marked in red).

One concern about patterns of lower fecundity at higher elevations is that the instability of these environments is likely to increase in response to climate change [12]. High elevations are thought to act as future climate refugia and many species are predicted to move upwards in elevation to escape increases in temperature [78]. However, weather whiplash and snow deluge events, or patterns of increasing severity in weather events, are expected to increase within montane environments, particularly at higher elevations [79–82]. This could result in larger and more severe snowstorms that not only impact overwinter survival but persist into the reproductive period, resulting in nest failures. We have included a graph of local snow depth data from weather stations located directly within our field site for visualization of these weather phenomena (figure 4). If high elevation individuals are already producing fewer offspring, populations may not be able to recover from climatic events that lead to individuals forgoing reproduction in especially challenging years [12]. Thus, it is critical to understand the basic patterns of reproductive output in wild populations, especially in environments most susceptible to climatic change as this may lead not only to insights into life history evolution but also help guide future management. It is important to mention that during the 12 years of the study we have never detected a nest failure that was related to inclement weather. The known failures in the system thus far have only been linked to mammalian depredation and parental disappearance or abandonment (personal observations of the authors).

In summary, our long-term study of resident songbirds suggests strong within-population differences in reproductive strategies associated with environmental differences across montane elevations over small spatial scales. Birds from higher elevations with harsher environmental conditions and a shorter breeding season appear to favour a strategy of producing fewer but higher quality offspring while chickadees at lower and milder elevations favour a strategy of producing more but lower quality young. These differences are likely tied to the fitness of these different localized groups within this resident species as they respond to local elevation-related conditions over time. Our results are inconsistent with the idea that birds simply adjust their reproductive output to the immediate environment within a given year. Future studies are needed to compare more such populations across a wider geographic area.

## Data Availability

All data for this study have been made available in a public repository [83].
